# Are baboons learning "orthographic" representations? Probably not

**DOI:** 10.1371/journal.pone.0183876

**Published:** 2017-08-31

**Authors:** Maja Linke, Franziska Bröker, Michael Ramscar, Harald Baayen

**Affiliations:** 1 Leibniz Institut für Wissensmedien, Tübingen, Germany; 2 Gatsby Computational Neuroscience Unit, University College London, London, United Kingdom; 3 Department of Linguistics, University of Tübingen, Germany; Plymouth University, UNITED KINGDOM

## Abstract

The ability of Baboons (papio papio) to distinguish between English words and nonwords has been modeled using a deep learning convolutional network model that simulates a ventral pathway in which lexical representations of different granularity develop. However, given that pigeons (columba livia), whose brain morphology is drastically different, can also be trained to distinguish between English words and nonwords, it appears that a less species-specific learning algorithm may be required to explain this behavior. Accordingly, we examined whether the learning model of Rescorla and Wagner, which has proved to be amazingly fruitful in understanding animal and human learning could account for these data. We show that a discrimination learning network using gradient orientation features as input units and word and nonword units as outputs succeeds in predicting baboon lexical decision behavior—including key lexical similarity effects and the ups and downs in accuracy as learning unfolds—with surprising precision. The models performance, in which words are not explicitly represented, is remarkable because it is usually assumed that lexicality decisions, including the decisions made by baboons and pigeons, are mediated by explicit lexical representations. By contrast, our results suggest that in learning to perform lexical decision tasks, baboons and pigeons do not construct a hierarchy of lexical units. Rather, they make optimal use of low-level information obtained through the massively parallel processing of gradient orientation features. Accordingly, we suggest that reading in humans first involves initially learning a high-level system building on letter representations acquired from explicit instruction in literacy, which is then integrated into a conventionalized oral communication system, and that like the latter, fluent reading involves the massively parallel processing of the low-level features encoding semantic contrasts.

## Introduction

It has recently been shown that Guinea baboons can be trained to discriminate between four-letter words (e.g., TORE, WEND, BOOR, TARE, KRIS) and nonwords (e.g., EFTD, ULKH, ULNX, IMMF) [1] simply by rewarding them for correct lexicality decisions. The number of words learned by the six baboons ranged from 81 to 307 (after some 60,000 trials), and they were reported to respond correctly to both novel words and nonwords with above chance performance. Further, these responses exhibited characteristic error patterns: nonwords that were more similar to words elicited more errors; and nonwords that were derived from words by transposing the middle two letters elicited more errors as compared to controls derived from words by letter substitutions [2]. These results have been taken to show that, even in the absence of other human linguistic knowledge, baboons are capable of learning a full orthographic code of letters and letter n-grams.

A deep learning, convolutional neural network (CNN) model has been proposed to explain the baboons’ performance, and the learning processes that underlie it [3]. The model successfully captures many aspects of average baboon learning across training trials. It also broadly simulated the baboons’ performance on lexical generalization, showing above chance accuracy for novel words and nonwords. Like baboons, the model also showed a trend to produce more word responses for nonwords with transposed middle letters compared to controls.

Convolutional neural networks (CNNs), which comprise a succession of alternating simple convolution and complex pooling levels, are considered to be particularly attractive models of visual processing because activations in their hidden layers have been shown to correlate with activations in areas in the ventral stream of primates and humans [4], findings that have been taken to suggest that like a CNN, visual processing can be characterised as a hierarchical system that extracts features from the environment at increasing levels of abstraction across its architecture. Analysis of CNN’s internal representations revealed that its performance relied on the development of “extremal units” that are selectively sensitive to position-specific letters, bigrams and trigrams, prompting the authors to conclude that the baboons’ reading abilities were mediated by a similar hierarchical processing system. And since the ventral stream of primates and humans share similar structure, [3] suggest that a similar system of hierarchical processing and orthographic codes underlies human reading.

However, the generality of this view is challenged by the results of a recent study [5] showing similar orthographic processing capacities in pigeons (columba livia) in a similar experimental setup. The pigeons successfully mastered between 26 and 58 words, and also exhibited both letter transposition and orthographic similarity effects, indicating that the avian visual system is also capable of learning to hierarchically represent and discriminate orthographically coded words. Notably, since pigeons’ brains lack a ventral pathway, this suggests that this structure itself may not be a necessary prerequisite for orthographic learning.

Further, although the reading abilities of both baboons and pigeons have been explained in terms of hierarchical processing and the extraction of orthographic representations, it is arguable that these presuppositions have been largely shaped by researchers’ introspective perspective on the process of human reading. The process of reading makes very different demands on orthographic processing than the simple lexical discrimination task completed by these animals. Whereas reading comprises a highly over-trained skill that integrates semantic and acoustic information with visual processing, baboons and pigeons are confronted with novel stimuli in which neither the letter strings as wholes nor even individual letters coded semantic or acoustic information, such that the task these animals were confronted with is better summarised as a discrimination task involving complex visual stimuli.

From this perspective, it is notable that experiments examining the hierarchical processing of visual stimuli (shapes made of shapes), manipulated for density and display size [6, 7] have shown that humans and baboons differ in their ability to perceive the higher level patterns in that baboons rely more on local, lower-level patterns. Further, baboons trained in discriminating same/different relationships perform worse when the number of discriminating cues is reduced [8].

Moreover, adult human subjects asked to identify a target stimulus from a set of distractor stimuli (visual search paradigm) show an asymmetry in response times: searching for a salient target between a set of distractors that violate expectations (closure, direction, e.g. elephants with feet up) yields longer response times than when the opposite is the case [9, 10], indicating that experience affects what is perceived at a local level. However, experiments aimed at examining asymmetry in responses on symmetric designs based on experience [11] show that here baboon performance again departs from that of humans. Humans respond faster to shared features within the category that was learned first, while only distinctive features appear to be strongly associated with the category learned subsequently. Unlike humans, baboons appear to process novel compound stimuli as a group of local features, rather than attending to the novel features exclusively [12]. Similar differences in category learning are found between children and adults [13]. Children, who (like baboons) lack critical brain structures to employ a resource preserving approach of attending to novel stimulus features exclusively [14], instead use all sensory cues present in their environment. Consequently, we can expect both the attentional resources of the baboons and their previous experience in learning to categorize sensory input to affect the representation of the stimulus features [15]. It is thus also notable that 4 of 6 baboons tested by [2] had previously participated in a visual categorization experiment prior to their lexical decision task, such that it is likely that not all of the stimuli in the latter experiment were equally available to all of the baboons.

So what lessons can be can be drawn from this body of research? The present study seeks to answer this question by characterising the information present in the task in the simplest, most biologically plausible way and establishing the minimal set of representational and computational capacities that are required in order to complete it. Stepping back from assumptions about hierarchical processing and orthographic codes, we sought to develop a model based on the most basic and objective principles. Beginning with a set of representations that can clearly be assumed to be shared by pigeons, baboons, and humans, we sought to examine the extent to which the performance of the baboons reported by [2] could be accounted for in the absence of any specific presuppositions about reading. We show that these baboons did not respond in a way that might be considered optimal, from the perspective of a model learning under perfect conditions. Further, we show that model performance improved markedly—especially at the level of the individual baboon—when we explicitly inform the model of the baboons’ behaviour on a trial by trial basis. Finally, our results question the necessity of sophisticated letter string representations or any kind of pre-linguistic visual systems, and instead offer an alternative explanation to hierarchical processing, providing a simpler and more parsimonious account of the animals’ behaviour.

## A wide learning approach

While the necessary characteristics of the system that subserves visual processing and its variations among species are still poorly understood, a large body of literature supports the finding that baboons, humans, and pigeons share cells that respond selectively to edges in the visual input [16–18]. Consequently, summarising visual information of letter stimuli by a set of edge sensitive features serves to represent the information content available to all three species at some point of the decision process.

In a first exploration of this approach, we successfully modeled visual word response in humans, finding that activations from a model trained on low-level visual cues for words from the written part of the British National Corpus explain 37,4% of variation in response latencies in the lexical decison data from the British Lexicon Project [19].

In the present study, we characterise stimuli by 14,476 discrete low-level visual features that approximate the encoding of orientation and contrast. They serve as input cues to a wide learning network [20, 21] based on the Rescorla-Wagner learning rule [22] which was trained to classify the input into words and nonwords.

Even this simplistic model predicts that performance should reach perfection after very little training. This indicates that the classification problem established by the stimulus material can be trivially learned by machine learning methods (see also [23–25]). However, these methods benefit from perfect learning conditions. In contrast, given their strong deviation from optimal learning, other factors must have affected the animals’ performance. The factors that presumably contribute to this discrepancy are hard to quantify, and include, among others, previous training experience [26], the motivations of the animals, their degree of attention, as well as their internal representation of the task [14]. Given that trial-by-trial baboon behaviour was clearly dominated by such factors, any model applied to the data should account for these effects in order to avoid the model from merely displaying the signature imposed by the interplay of a model’s hand-crafted architecture and a specific input sequence. By contrast, the Rescorla-Wagner model as well as deep neural networks are, in their classic application, data independent models, whose performance is driven by the stimuli only.

It thus follows that a mechanism is required that allows a learning model to be informed by both the task environment and the observed behavior of the animal. The behavior of individual baboons followed clear trends over the course of the experiment, and consequently it follows that to model individual baboon behavior, learning should be informed in a trial-by-trial fashion to yield more reliable insight into the inner workings of the actual process. Typically such methods include inducing noise when updating weights, or the optimisation of free model parameters in the pooling and convolution steps as was done in case of the CNN. However, none of these methods provides a principled approach to modelling the specificities of trial-by-trial behaviour as their effects are unspecifically distributed over the entire set of stimuli and trials instead of tailored to the actual temporal dynamics. For this reason, we applied an alternative mechanism inspired by the fact that learning not only happens in the presence of explicit feedback, but can be also reinforced by an agent’s own decision in the absence of feedback [14, 27, 28]. This amounts to a two-stage update of the model’s weights in learning. First, the model is correcting its predictions by the actual lexical decision made by the baboon, in order to account for imperfect learning. Second, the model is updated by the feedback that is received as a matter of course from the experimental apparatus. That is, this two-step process enables us to examine a possible explanation of imperfect learning while exploiting a training signal that remains faithful to the information that can be objectively extracted from the experimental trial structure.

Technical details about the implementation of the model are available in the supporting information section.

## Analysis

**Fig 1:**
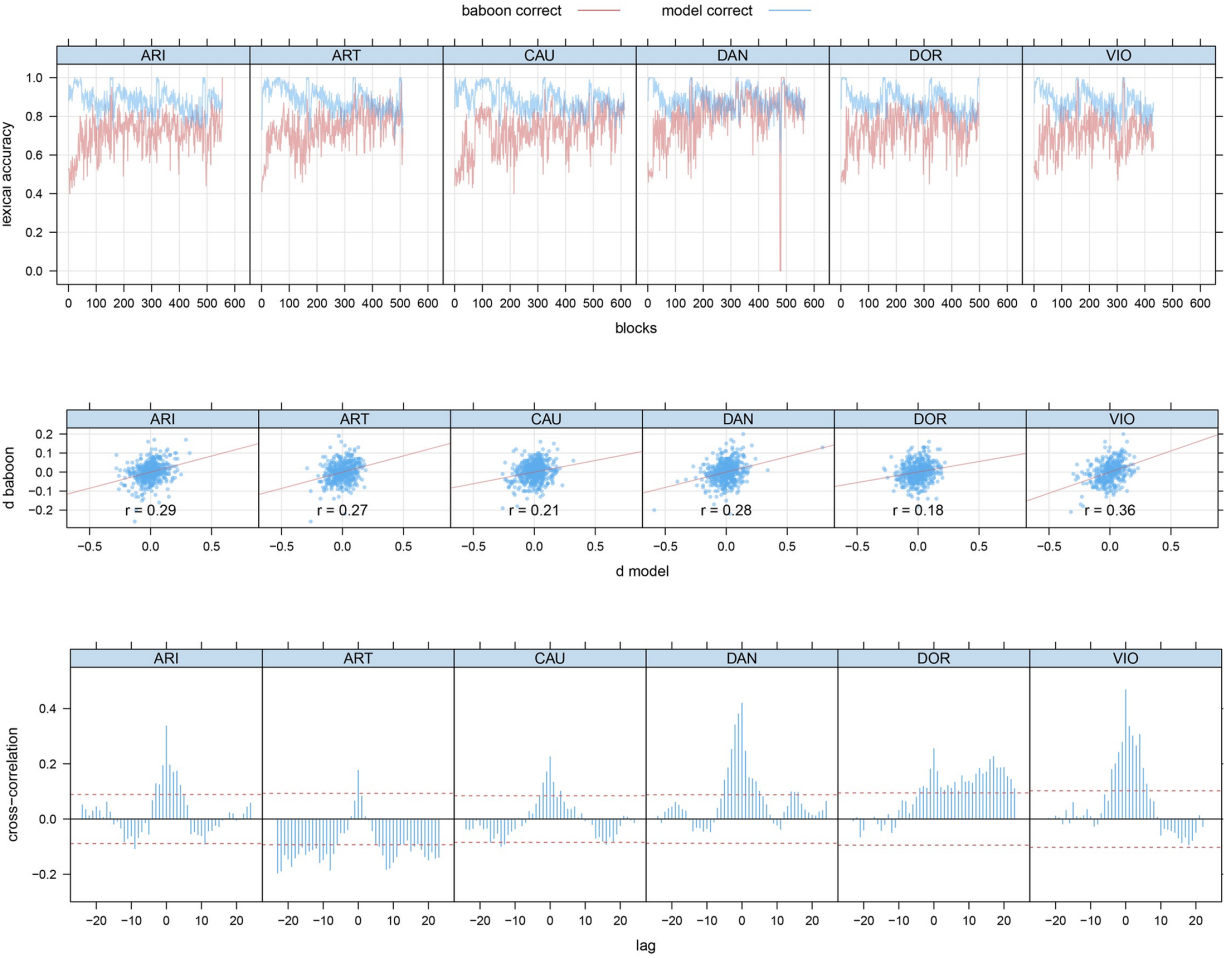
Baboon and model accuracy. Upper panel: discrimination accuracy for bins of 100 trials. Second panel: Change in accuracy for successive bins (all *p* ≤ 0.0001). Third panel: crosscorrelations. As initial baboon behavior is idiosyncratic, panels 2–3 are based on analyses excluding the first 5000 trials.

We trained six networks, one for each baboon, updating the learning weights of each model on each trial with exactly the same stimulus sequence as presented to the individual baboons. Fig 1 summarizes results for baboon and model accuracy. The top panel shows that the model still performs the discrimination task with greater accuracy. Importantly, block-to-block changes in accuracy for baboons were matched by similar changes in model accuracy (second panel). Significant crosscorrelations at lags other than 0 (third panel) provide further support that the model captures significant aspects of baboon performance.

**Fig 2:**
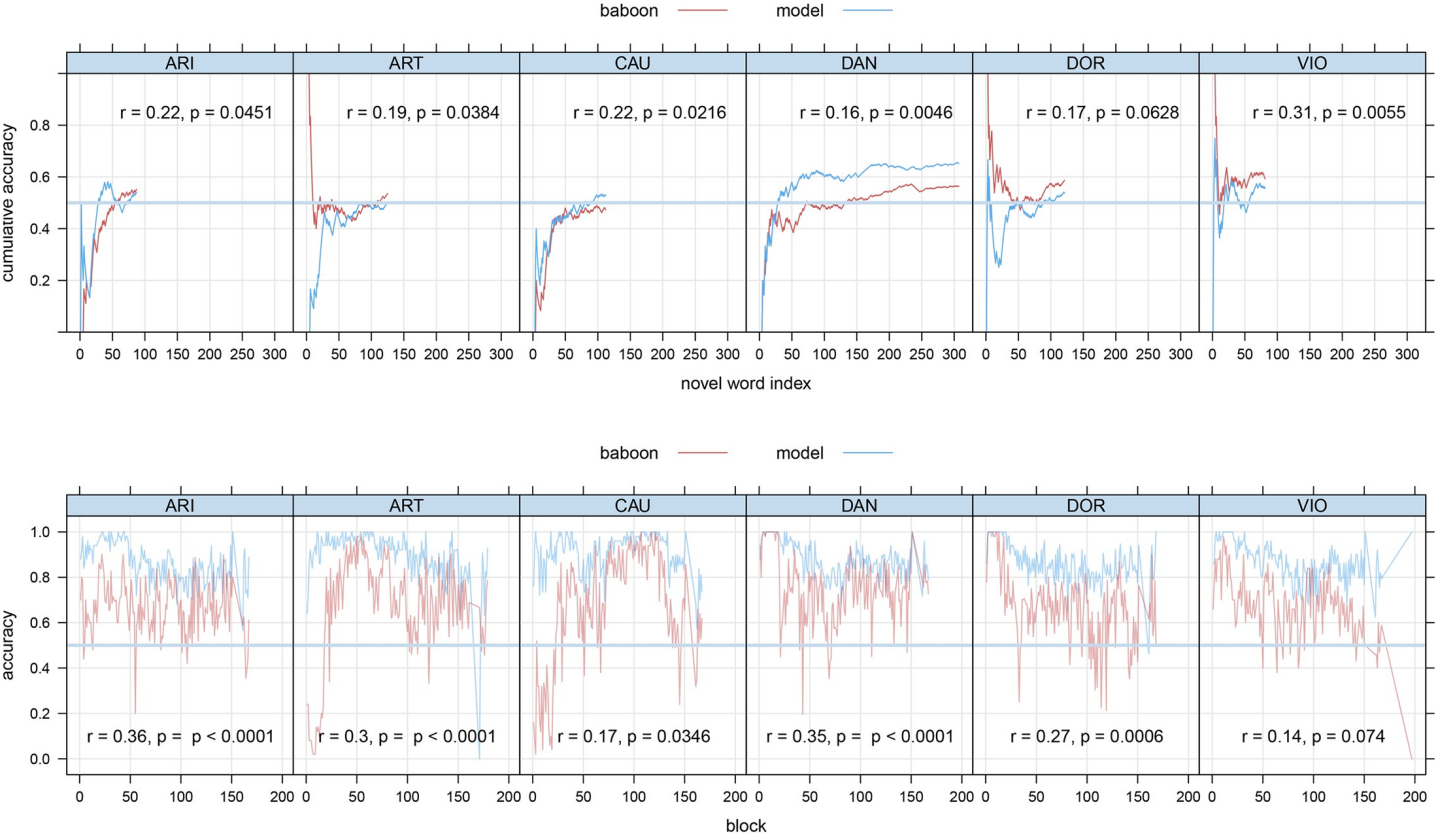
Generalization in baboons and model at first encounter of a novel word (upper panel) or nonword (lower panel). Upper panels show cumulative accuracy as novel words are encountered. Lower panels show accuracy on first encounters of nonwords by blocks of 100 trials. Correlations are Spearman correlations based on the empirical first derivatives of baboon performance and model predictions.

Generalization performance on novel words and nonwords is summarized in Fig 2. As novel words are very sparse, we graph cumulative accuracy for words against novel word index (top panel). The extent to which curves extend to the right reflects the number of words learned: Baboon CAU mastered 112 words, whereas baboon DAN learned 307 words to criterion. Although overall baboon (and model) accuracy is above chance on novel words, the performance of, e.g., baboon CAU reveals a strong nonword decision bias that reduces in the course of learning, but leaves performance of this baboon only at chance by the end of the experiment: accuracy is exactly at 0.5 on the last 10,000 trials. For DAN, performance over the last 10,000 trials is slightly above chance (44/80 = 0.55), and shows an upward trend that is lacking for CAU (Fig 2). Spearman correlations of the first derivatives of baboon and model learning curves again indicate that model predictions shift up and down in tandem with baboon performance.

Performance on novel nonwords is shown in the bottom panel of Fig 2. Baboon CAU shows an initial increase in accuracy, followed by a decrease, resulting in a final overall accuracy for novel nonwords close to chance. DAN, by contrast, shows more consistent behavior. First derivative tests indicate that the models are predicting the ups and downs in baboon learning to some extent also for the subset of nonwords.

In order to compare the performance of the present discrimination network with the CNN of [3], we extracted deep learning accuracy from Fig 2 in [3] using digitizeR [29]. The correlation between the first derivative of baboon and model performance functions does not reach significance (*r* = 0.39, *p* = 0.0639, *r*_*s*_ = 0.22, *p* = 0.1762), and there were no significant cross-correlations. This further underlines that it is not sufficient to simply assume a learning model purely driven by the stimulus input, but that baboon behaviour was significantly influenced by the internal state of the baboon.

**Fig 3:**
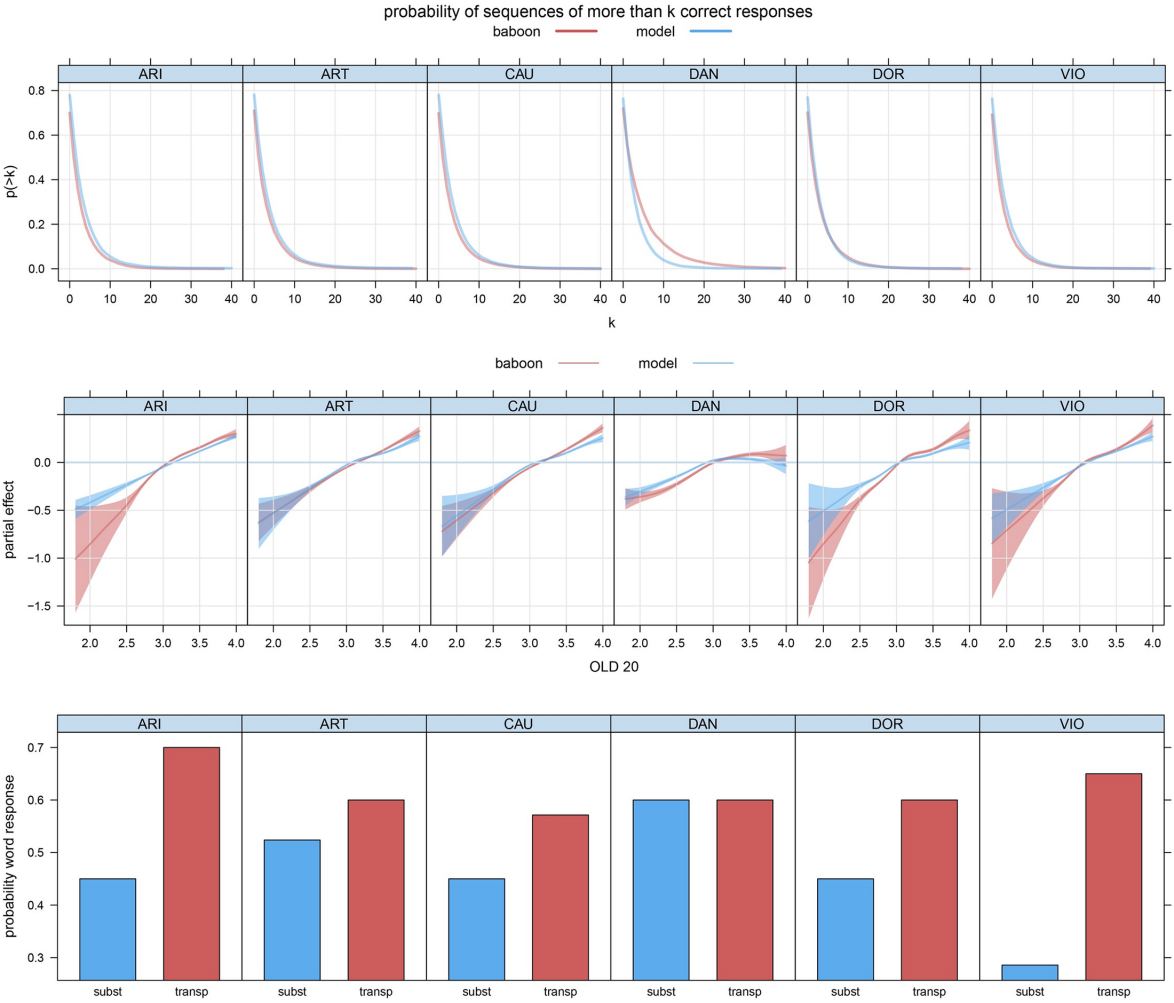
Upper panel: probability of sequences of more than *k* correct responses. Center panel: partial effect of OLD20 on nonword accuracy in a generalized additive mixed model with by-baboon factor smooths for trial, for observed baboon performance (red) and model predictions (blue). For both baboons and model, greater dissimilarity from words (greater OLD20) predicts higher nonword accuracy. Lower panel: Probability of word response predicted by the models for the baboons, subcategorized by type of nonword. Transp: nonword obtained from a word by transposition of the center letters; subst: nonword obtained from a word by exchanging a vowel for a vowel or a consonant for a consonant.

To move away from dependency on bin size, the top panel of Fig 3 shows the probability of observing sequences of more than *k* correct responses. Again, model performance closely resembles baboon performance. In addition to characterising overall performance, we tested whether our model could account for the specificities in the error patterns observed for the stimuli. The center panel of Fig 3 shows that nonwords that are less similar to words, as indexed by a higher value of the OLD20 neighborhood measure [30], are more likely to elicit correct nonword decisions. Here, the discrimination models provide tight predictions for baboon performance.

In a follow-up experiment [2], baboons were more likely to make erroneous word responses to nonwords obtained from words by transposition of the center letters, as compared to nonwords obtained by substituting vowels or consonants. To explore the basis of this effect, we constructed multiple sets of stimuli following the instructions in [2], including transposition and substitution nonwords and visually similar and dissimilar targets. The results from 10 simulation runs revealed a similar trend to that reported by [2]. For visual similarity targets, no effect was present ($\hat{\beta }=-.09,Z=-1.14,p=0.2533$). Thus, the predictions of the model and the baboon behavior are consistent with findings showing that illiterate humans [31] and baboons [8] have difficulties discriminating visual stimuli when only small alterations are made to their internal structure. Furthermore, as illustrated in the bottom panel of Fig 3, which depicts one simulation run, across runs the baboon models predicted more erroneous word responses for nonwords derived through transposition ($\hat{\beta }=-0.77,Z=-3.67,p=0.0002$). We should note that this effect was severely attenuated for 2 of the baboon models (${\hat{\beta }}_{ARI}=-0.22$, ${\hat{\beta }}_{A}RT=-0.25$). (We should further note that these follow-up simulations were not informed by double learning, because we did not have the baboon trial to trial performance at our disposal.)

The variability in model performance is hardly surprising given that we know that effects of letter transpositions are reduced in sequences of pseudoletters or letters from an unfamiliar alphabet [32] and that their strength varies depending on whether the transposed letters are vowels or consonants [33]. Importantly, although high tolerance for letter-position pertubation is displayed by readers of English and other languages with high orthographic redundancy, this effect does not generalize to a language like Hebrew [34, 35], where information is packed more densely. While English readers can make sense of words with letter transpositions and words with omitted vowels, readers of Hebrew fail to do so for words with Semitic root structure. Previous modeling using discrimination learning correctly predicts letter transpositions to have detrimental effects in Hebrew [36], but not in English. After transposing letters, sufficient low-level orthographic cues remain in English to support the intended readings, whereas in Hebrew critical cues are absent and recognition collapses.

Following on from this line of reasoning, an analysis of visual cue weights from the simulations reported here revealed that cues positioned at the first letter slot (*n* = 2586, *Mdn*_*w*_ = 0.00022) tended to provide stronger support for word/nonword responses, as compared to the slots in the middle of the word (*n* = 2246, *Mdn*_*w*_ = 0.00012). Thus, these results further suggest that the baboons’ sensitivity to stimulus type is simply a function of the stimulus set that they were exposed to (in the same way that the behavior of English and Hebrew speakers is a function of the stimulus set they have been exposed to). Accordingly, it cannot be taken as evidence for the existence of representational units as [2] claim.

So far, we have shown that a foundational learning algorithm [22, 37–39] implemented with just a single free parameter the value of which was fixed beforehand on the basis of earlier results on modeling human lexical decision behavior [40], succeeds surprisingly well in predicting baboon lexical decision behavior. The model predicts the ups and downs in baboon accuracy, it generalizes, produces more nonword decisions for nonword stimuli that are less similar to words, and it reproduces the letter transposition effect.

Based on the selected model architecture, the CNN developed extremal units that showed sensitivity to letter bigrams and trigrams. It was claimed, furthermore, that baboons did not base their decisions on holistic word-shape information [2, 41], the idea being that it would be computationally more efficient to solve shape-invariance at the level of individual letters and letter combinations than at the level of whole words. However, the deep learning network could have been set up with further layers and with more extremal units to allow word-specific representations to emerge. The question therefore remains to what extent the behavioral data themselves support letter n-grams and holistic word units as explanatory variables. A danger inherent in imputing holistic lexical representations to the baboon brain is that properties of the input to which the baboon is exposed can be reflected in the baboon’s behavior, without mediation in the brain by (sub)lexical representations. And indeed, our ability to predict baboon behaviour based on simple low-level visual features without any explicit representation of letter strings, suggest that the latter are not necessary components in order to solve this discrimination task.

We therefore analyzed the trial-to-trial choice behavior of the six baboons with the generalized additive mixed model [42–48]. For each baboon, logistic gamms were fitted to the word data, each using a thin plate regression spline predicting choice behavior from experimental time (training trials), but with different random-effect factors. One model included word stimulus as random-effect factor (e.g., TORE), a second model included the first and second trigrams (e.g., TOR and ORE) as random-effect factors, and a third model included the three bigrams (e.g., TO, OR, RE) as random effects. Each model included an autoregressive AR(1) process in the errors, with correlation parameters ranging across baboons from 0.066 to 0.110. A total of 36 tests for significance of random effects [48] (for each of 6 baboons, one test for word, two tests for trigrams, and three tests for bigrams) were evaluated with a Bonferroni-adjusted *α* = 0.05/36 = 0.0014. A random effect of word was supported for each baboon, the first trigram was never significant, the second was significant for 1 baboon, the first bigram was significant for 5 baboons, the second for 2, and the third for 3. However, the goodness of fit of the 6 models with bigrams as random effect was worse than that of the model with word as random effect for 5 out of 6 baboons, while requiring fewer degrees of freedom.

## Discussion

**Fig 4:**
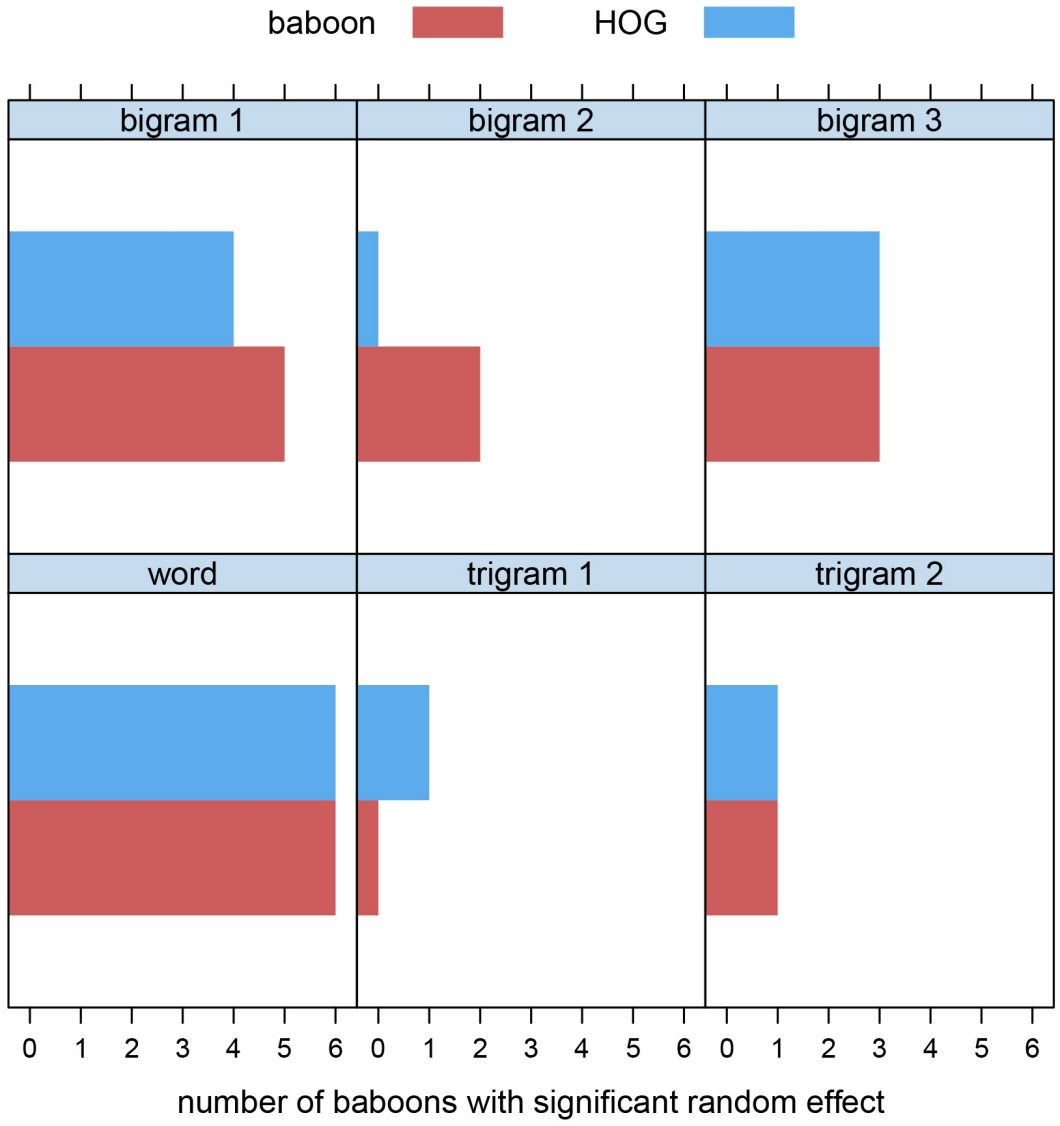
Number of baboons for which random effect factors (words, bigrams, trigrams) were significant according to the test described in [48], for baboon responses and responses predicted by the model.

These results cast new light on the claim [2, 41] that the word is an irrelevant unit for understanding baboon discrimination behavior. If one takes the results of the statistical analysis at face value, one sees strong evidence that one could take as support for internal representations of orthographic words. In fact, a simple objective interpretation of the statistical analysis suggests that orthographic word representations better explain baboon behavior than letter pairs and letter trigrams. However, it should be noted that this is hardly surprising since both words and letter pairs are objective parts of the input stimuli. Despite the fact that our models’ internal representation contains neither word forms nor letter pairs or letter trigrams, when we apply the exact same statistical analysis to its predictions, we find exactly the same patterns of evidence (Fig 4) that traditionally are taken to reveal internal representations of word forms, letter pairs, and letter trigrams. That is, although none of these levels of orthographic representation is present in our model, it yields the same patterns of evidence that have been taken to support mediation by orthographic representations at one level or another.

Instead of assuming that baboons and pigeons learn lexical representations [3, 5], the most straightforward and parsimonious explanation is that both baboons and pigeons optimize their chances of reward not by constructing a hierarchy of lexical units, but rather by making optimal use of the strengths that come with a massively parallel processing system driven by low-level visual features. Furthermore, models suggest that even human behaviour observed in visual detection tasks can simply be predicted from population activity in the primary visual cortex [49]. This does not rule out the possibility that visual processing proceeds in a hierarchical manner which is necessary to solve tasks in which low-level features are not sufficient in order to make robust decisions. However, skilled fluent reading in humans may likewise be grounded in massive parallel processing from low-level visual features to semantics. From this perspective, the function of letter by letter learning during the acquisition of literacy is a means for bootstrapping into the subliminal yet powerful low-level parallel processing systems that we share with baboons and pigeons.

**Fig 5:**
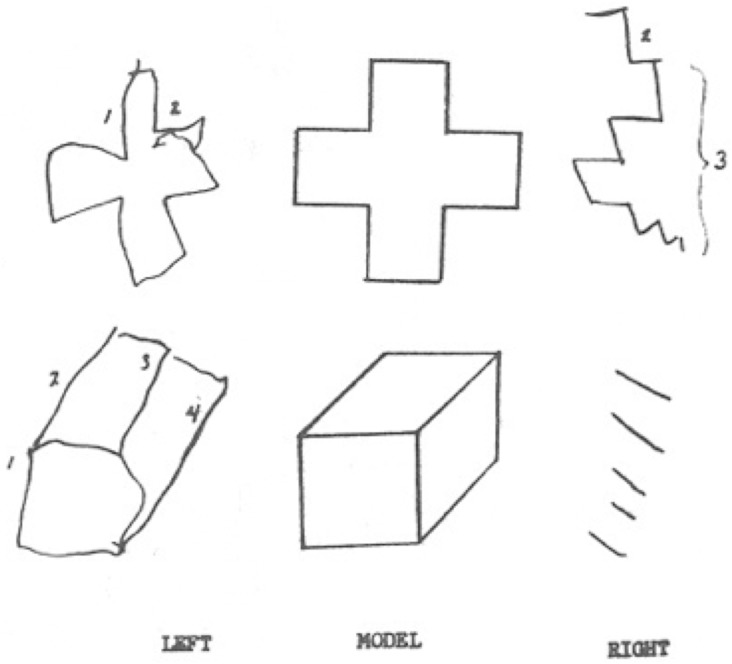
Left-hand and right-hand model drawings of a right-handed 13 year old patient 5 months following a cerebral commissurotomy [50].

Finally, we should acknowledge that although our model captures many aspects of the baboons’ behavior, primate brains have a complex architecture comprising many subsystems such that it is unlikely that any behavior, including the present baboon behavior, is the product of just one of these. This point is beautifully illustrated by Fig 5, which depicts a rendering of two simple stimuli as presented independently to either hemisphere of a split brain patient [50]. For this patient, “seeing”, a cross or a box appears to be a function of the right hemisphere who’s internal representation seems to bear some resemblance to reality, as illustrated in Fig 5 by the drawings to the left. By contrast, the rendering to the right would appear to suggest that when these stimuli are represented in the left hemisphere, which subserves naming, sorting, and discrimination, what can be inferred about this patient’s internal representations bears little resemblance to what the right hemisphere perceives, or indeed, what appears to be objectively in the environment. Of course, behaviorally, someone with an intact corpus collosum would see a cross and a box, and draw a cross and a box. What Fig 5 suggests is that no single system subserves these abilities. Similarly, it is unlikely that reading tout court is entirely subserved by the ventral pathway or any other single neural structure. Consistent with this, our simulation results suggest not that there are no orthographic representations, but rather that the systems and representations involved in reading are likely to be similarly distributed. Accordingly, depending on context and task, numerous sources of information at various levels of abstraction can be expected to contribute to the process, including direct associations between low-level visual features and higher-level knowledge. Indeed, the very fact that humans, baboons, and pigeons can all learn this lexical discrimination task seems to underline this point.

## Supporting information

S1 Methods (PDF)
