# Supplementary material for: Are baboons learning "orthographic" representations? Probably not
Source: PLoS One. 2017 Aug 31;12(8):e0183876. doi: 10.1371/journal.pone.0183876 (PMC5578497; doi:10.1371/journal.pone.0183876)
Supplement: S1 Methods — (PDF) [file pone.0183876.s001.pdf]

Are baboons learning "orthographic" representations? Probably not.

Maja Linke[1, *], Franziska Bröker[2], Michael Ramscar[3], Harald Baayen[3]

1 Leibniz Institut für Wissensmedien, Tübingen, Germany
2 Gatsby Computational Neuroscience Unit, University College London, London, United Kingdom
3 Department of Linguistics, University of Tübingen, Germany

*** m.linke@iwm-tuebingen.de**

Supporting Information

S1 Methods

The networks used to simulate baboon behavior were trained with the simplest possible error-driven learning rule known from the animal learning literature, proposed in 1972 by Rescorla and Wagner [1]. We constructed, for each baboon separately, a network with word and nonword output nodes, and with as input units, discrete gradient orientation features derived from a simplified version of the histogram of oriented gradients (HOG) feature descriptor first proposed by [3]. The choice for HOG-based features as input is motivated by studies indicating that vision primitives encode contrast and orientation [4–7]. HOG features are descriptors of low-level features of an image with respect to gradient magnitude, gradient orientation, and spatial location. The algorithm computes magnitude of gradient orientation in densely distributed locations of an image. For this, we divided stimulus images in a grid containing non-overlapping cells of fixed size. We used a grid of 10 by 4 such cells for the four-letter words presented to the baboons. For each cell, we extracted the gradient magnitude at each pixel contributing a weighted vote to 9 gradient orientation bins of 20 degrees each. Each pixel also adds a weighted contribution to the four neighbouring orientation bins and the respective bins of four neighbouring cells. The resulting histograms values were normalized (with the regularized L2-norm) across cells, yielding for each stimulus image a gradient-based vector with $40 \times 9 = 360$ values in $[0, 1]$. To capture locality and orientation, we add to each value a head argument encoding the feature descriptor index, representing the cell position and gradient bin. The 8139 distinct word and nonword stimuli of the baboon study generated a total of 14,476 unique local gradient orientation (LGO) features.

For each baboon, a separate network was trained on the sequence of stimuli in exactly the order presented to that baboon in the experiment. Weights were updated with the Rescorla-Wagner learning rule. This rule specifies the change Δw_{ij} in the weight on the connection from LGO input node h_i to lexicality output node $o_j, j = 1, 2$, taking into account all LGO features h_m in the set H of LGO features present in the input, as follows:

$$\Delta w_{ij} = \begin{cases} 0.001 \cdot (\mathrm{I}_{[o_j \text{ is reinforced}]} - \sum_{m \in H} w_{mj}) & \text{if } h_i \in H \\ 0 & \text{otherwise} \end{cases} \qquad (1)$$

The learning rate, the only free parameter of the model, was held constant at 0.001 across baboons, following previous results indicating this learning rate to be optimal for the modeling of human lexical decision [2].

At each trial, the Rescorla-Wagner learning rule was applied twice. The first update of the weights was driven by the baboon's own response. A word response led to reinforcement of the weights to the word output node and weakening of the weights to the nonword output node, whereas a nonword decision led to reinforcement of the weights to the nonword node and a downward adjustment of the weights to the word node. Whereas in a typical lexical decision task human subjects are not provided with feedback on the accuracy of their decisions, the baboons did receive such feedback. Food was dispensed for correct responses, and a screen flash without food indicated the response was incorrect. The consequences of this feedback were modeled by applying the Rescorla-Wagner learning rule a second time, but now with lexicality (word versus nonword) driving the updating of the weights. Correct responses led to strengthening of the weights to the word c.q. nonword nodes, whereas incorrect responses led to downward adjustments.

For each trial t, prior to the updating of the weights, we calculated the bottom-up support provided by the LGO features in the input for the word and nonword responses. Bottom-up support was estimated by summation of the weights ($w^{(t)}$) at t on the connections from the LGO features in the input to the word and nonword output nodes o_{word} and o_{nonword}. This resulted in two activations,

$$a(\text{word}, t) = \sum_{m \in H} w^{(t)}_{mo_{\text{word}}}$$

for the word and

$$a(\text{nonword}, t) = \sum_{m \in H} w^{(t)}_{mo_{\text{nonword}}}$$

for the nonword response. A word response was generated at trial t when $a(\text{word}, t) > a(\text{nonword}, t)$, and a nonword response otherwise.

In the early phase of the experiment, baboons had a strong bias for either nonword responses (baboons DAN, DOR, VIO and to some extent ARI) or for word responses (baboons ART, CAU), see Fig 2 bottom panel. Therefore, the correlational analyses in Figs 1 and 2 excluded the first 5000 trials from consideration. For baboon ART, this cut-off point is too early (upper panel Fig 2, lower panel), resulting in outliers violating normality and giving rise to artefactual negative crosscorrelations (bottom panel Fig 1).

References

1. Rescorla RA, Wagner AR. A theory of Pavlovian conditioning: Variations in the effectiveness of reinforcement and nonreinforcement. In: Black AH, Prokasy WF, editors. Classical conditioning II: Current research and theory. New York: Appleton Century Crofts; 1972. p. 64–99.

2. Bitschnau S. An exploration of computational learning algorithms: A closer look at the Rescorla-Wagner model and the Danks equilibrium equation in language processing; 2015. BA thesis Cognitive Science, University of Tübingen.

3. Dalal N, Triggs B. Histograms of oriented gradients for human detection. In: 2005 IEEE Computer Society Conference on Computer Vision and Pattern Recognition (CVPR'05). vol. 1; 2005. p. 886–893.

4. De Valois RL, Yund EW, Hepler N. The orientation and direction selectivity of cells in macaque visual cortex. Vision research. 1982;22(5):531–544.

5. Kapadia MK, Ito M, Gilbert CD, Westheimer G. Improvement in visual sensitivity by changes in local context: parallel studies in human observers and in v1 of alert monkeys. Neuron. 1995;15(4):843–856.
6. Burr DC, Morrone MC, Spinelli D. Evidence for edge and bar detectors in human vision. Vision research. 1989;29(4):419–431.
7. Watt R, Morgan M. The recognition and representation of edge blur: evidence for spatial primitives in human vision. Vision research. 1983;23(12):1465–1477.
